# Life around the North Water ecosystem: Natural and social drivers of change over a millennium

**DOI:** 10.1007/s13280-018-1028-9

**Published:** 2018-03-08

**Authors:** Kirsten Hastrup, Astrid Oberborbeck Andersen, Bjarne Grønnow, Mads Peter Heide-Jørgensen

**Affiliations:** 1 Department of Anthropology, University of Copenhagen, Øster Farimagsgade 5, 1353 Copenhagen K, Denmark; 2 Department of Learning and Philosophy, The Techno-Anthropology Research Group, Kroghstræde 3, Building 4249, 9220 Aalborg Ø, Denmark; 3 The National Museum of Denmark, Frederiksholms Kanal 12, 1220 Copenhagen K, Denmark; 4 Greenland Institute of Natural Resources, c/o Greenland Representation, Strandgade 91, 2, Postbox 1915, 1016 Copenhagen K, Denmark

**Keywords:** Drivers of change, Ecosystem dynamics, North Water, Smith Sound, Social–ecological transformation

## Abstract

The formation of the North Water in Smith Sound about 4500 years ago, as evidenced by the establishment of bird colonies and human presence, also initiated a long-term anthropogenic agent as part of this High Arctic ecosystem. Different epochs have influenced the human occupation in the area: immigration pulses from Canada and Alaska, trade with meteorite iron throughout the Arctic, introduction of new technologies by whalers and explorers, exploitation of resources by foreigners, political sequestration, export of fox and seal skins and later narwhal products, and recently fishing. Physical drivers in terms of weather and climate affecting the northern hemisphere also impact accessibility and productivity of the ecosystem, with cascading effects on social drivers, again acting back on the natural ecologies. Despite its apparent isolation, the ecosystem had and still has wide ranging spatial ramifications that extend beyond the High Arctic, and include human activity. The challenge is to determine what is internal and what is external to an ecosystem.

## Introduction

**Fig. 1:**
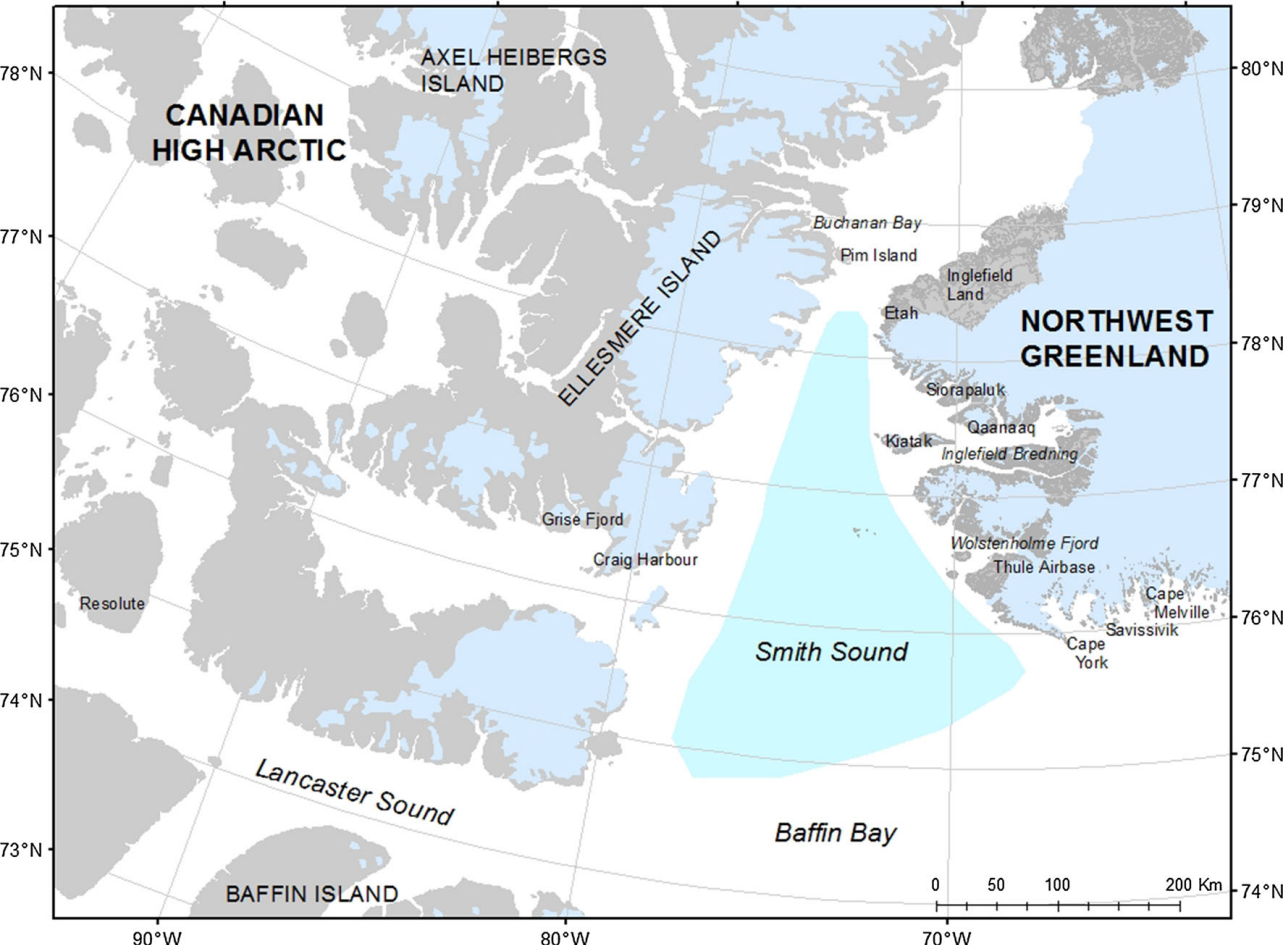
Map of the region dealt with in this article, with relevant place names. The ‘Thule Region’ referred to in the text, covers the coastline from Cape Melville to Inglefield Land where descendants of Thule Culture immigrants (from c. 1250 AD onwards) have lived as hunters. The marked area in Smith Sound shows the extension of the North Water

Human life in the Thule Region, Northwest Greenland (Fig. 1), always depended on the North Water polynya and its ability to sustain predictable animal populations, which could be hunted by coastal communities. The relationship between the polynya and the people was not one of passive harvesting of whatever was available, but a dedicated attempt at exploiting particular species perceived as necessary or valuable within the social framework. This point of departure challenges easy notions of the ecosystem as a closed system of interdependence between species or between people and their resources.

Analysing the Smith Sound region as an ecosystem, within which human practices—their movements, their needs, their aspirations, their travels—matter, allows us to sense the constant process by which the system’s boundaries are refashioned. The plasticity of the ecosystem is made by different temporalities: the fabrication of a kayak, the seasonal migration of birds, the movements of sea and land mammals, changing climates, struggles for sovereignty and power, shifting commercial and use value of living resources, and the networks of places within or beyond the ecosystem, among many others. In other words, we see ecosystems as “products of culture and power, as much as temperature and rainfall” (Hoag and Svenning 2017, p. 28).

This interdependence will be demonstrated by focusing on particular moments in the history of human habitation in the region, from c. 1250 AD to present. These moments do not add up to a ‘total history’ of the long-term development; they are examples in a larger vision of the ecosystem as affected by practices and interests of people living within and beyond the region. With such practices as drivers of social and ecological change, the challenge is to determine the extension of the ecosystem and how its spatial plasticity can be addressed while still analysing it as a ‘system’.

The North Water is part of a High Arctic ecosystem touching on the coasts of both Northwest-Greenland and Ellesmere Island on the Canadian side of Smith Sound (Fig. 1). For the present-day hunters in the region, it is almost exclusively the coastlands east of Smith Sound that are considered hunting grounds, but this was not always so. People migrated into the region from the west, and when looking at the dispersal of resources, it makes sense to include both the western and the eastern sides of the Smith Sound, even if they are now separated by national boundaries. In spite of these boundaries, hunters of the Thule region have visited Ellesmere Island also in modern times (Vibe 1948). While the eastern side of Smith Sound was always more productive and offered more game, excursions to Ellesmere Island served particular purposes at various points in time. Going there was a distinct spatial practice, tightly interwoven with social, technological, and political aspects of access to particular animal resources.

The ecosystem, as approached here, incorporates diverse climatic, physical, and biological, as well as social and historical factors. Humans contribute to the dynamics through their activities. Looking back in time on the different waves of human occupation of the High Arctic region around Smith Sound we note how they left their own distinctive marks on the environment (Schledermann and McCullough 2003; Grønnow and Sørensen 2006). In historical times (from the 19th century onwards), explorers and scholars have added knowledge about human life in the region and about the hunting practices by which people have survived—sometimes barely, as was the case in the first half of the 19th century (Kane 1856; Hayes 1866). In turn, this led to intensification of the walrus (*Odobenus rosmarus rosmarus*) hunt after the introduction of rifles in the region in late 19th and early 20th centuries (e.g., Peary 1898), and underpinned by recent biological studies, testifying to dramatic reduction of walrus abundance in this period (Born 2005). Lately, another human contribution to the upheaval of the ecosystem is the recent (anthropogenic) global warming which is amplified in the High Arctic (AMAP 2017).

The main thrust of this paper is a comprehensive history of the Smith Sound ecosystem including the human presence—a history that emphasises how different historical epochs emerge both through changing opportunities and through changing perceptions of values. Intending to write an ecosystem history is a bold task, one that unsettles the categories of established scientific disciplines, and faces the challenge of weaving together descriptions and arguments across multiple temporalities. In times of increased human-induced environmental change in the Arctic and elsewhere, it is important to develop new analytical tools that show how human and environmental histories cannot be separated. Humans drive environmental change, as do climatic factors, if at different scales at different points in time. The plasticity of the ecosystem examined here consists of social practices, economic activities, and comprehensive environmental changes to which living resources, ice conditions, and human beings respond. Writing occurrences forth as moments, epochs, phases, we intend to curb the risk of getting caught in a specific mode of chronological writing, presumably adding up to a complete history, and instead emphasise that different temporalities, past and present, are ‘thrown together’ (Massey 2005) in the make-up of the Smith Sound ecosystem, including its human agents.

## Early history of the ecosystem: Arrivals

The Smith Sound ecosystem is of relatively recent origin, having emerged only after the end of the glacial retreat 8000 years ago, that gradually opened the Arctic seas and allowed wildlife and people to populate the area. Large little auk (*Alle alle*) colonies formed in the area some 4500 years ago (Mosbech et al. 2018); this presupposed that a polynya had formed creating productivity that could support copepods which eventually could sustain the little auk colonies (Davidson et al. 2018). The polynya offered conspicuous concentrations of marine game that could support the human migrations to the area as evidenced from the archaeological traces of the earliest human habitations in the region from about the same time as the arrival of the little auks (Grønnow 2017).

The first palaeo-Eskimo settlers in the region were followed by later waves of immigrants interrupted by long periods with no presence of humans (Grønnow and Sørensen 2006). The latest known major immigration was that of the Thule Culture (c. 1250 AD), hailing from the Bering Strait and moving east along the Arctic coast and within a generation or so entering Northwest Greenland. They brought the *umiaq* (the big communal skin-boat), the kayak, and the dog-sledge into Greenland (McGhee 1997; Schledermann and McCullough 2003). These technologies made whaling possible, and from the ruins excavated in the region, it is likely that at least in the early phase, the Thule Inuit in Greenland were whalers; they had a social structure facilitating communal whale hunting and technology for catching bowhead whales (*Balaena mysticetus*) (Holtved 1944, p. 73; McCullough 1989).

From the early phase of Inuit settlement in the region, there is ample archaeological evidence of contacts between the settlers and people far away. Metals in the shape of meteoric iron from the southern part of the Thule Region and native copper from the central Canadian Arctic were traded between Inuit groups in the Eastern Arctic, walrus ivory and perhaps other commodities were exchanged with (or taken from) Norse boat teams travelling from the Eastern and Western Settlements to the High Arctic. This shows that the Inuit societies in the Smith Sound region periodically were part of much larger networks like the palaeo-Eskimo societies before them (Schledermann 1990; Sørensen 2010, 2012). It also shows how the boundaries of the ecosystem are difficult to determine. In general, humans as well as migratory animal species, biomass, and mineral resources have entered and left the space of the ecosystem, shaping and altering it, in different times of history.

The onset of the Little Ice Age brought economic and demographic changes to people living in Smith Sound. Early in the 15th century, a prominent expansion of the range of the Inuit population in North Greenland took place as groups of Early Thule Culture affiliation migrated via Smith Sound north around Peary Land and into Northeast Greenland (Sørensen and Gulløv 2012). The Thule Culture groups that remained in the North Water area during the Little Ice Age changed in many respects: the *umialik*-institution (the hierarchical social organization around the whale hunting teams) disappeared, winter settlements apparently became smaller and spread into hitherto unsettled areas in the deep fjords, where new resource spaces with a focus on walrus, small whales, and ringed seal (*Phoca hispida*) appeared. The archaeological evidence from this period is sparse and understudied, but it is safe to say that during the culmination of the Little Ice Age during the 16th–18th centuries, the population of the Smith Sound area became increasingly isolated and diminished considerably—perhaps to a point of (near-) extinction (Schledermann and McCullough 2003).

In the process, the Thule Culture changed; bowhead whales were no longer a prominent game for the people remaining in the region. Stranded whales may still have played some role in their economy; it is impossible to distinguish between bone material from hunted or stranded whales (McCartney 1984, p. 101). Another, and probably more important, because more stable, resource that has been available since the earliest times of the Thule Culture was the walrus, present on both sides of Smith Sound (Gotfredsen et al. 2018). Recent analyses of the walrus movement in Smith Sound show that there is probably just one stock (known as the Baffin Bay or the North Water stock), prevalently wintering on the Greenlandic side, while spending summer on the Ellesmere side in Jones Sound, Lancaster Sound and Buchanan Bay and adjacent areas (Stewart et al. 2010; Heide-Jørgensen et al. 2017). This is probably a long-term pattern related to the different sea ice formations and bathymetry of the two sides of Smith Sound—warmer and likely more productive on the Greenlandic side (Born et al. 2017, p. 89). In addition to providing meat for humans and dogs, walrus ivory was an important resource for trading. It has been suggested that, “exploitation by man has been the main driving force behind the dynamics (and severe reduction) in Greenland walrus populations” (Born 2005, p. 4). In Northwest Greenland, this may be true for the past 100 years, but seems unlikely until early 20th century, where human presence in Smith Sound was sparse and walruses abundant.

During the Little Ice Age (c. 1350–1800), the human migration across Smith Sound (nearly) came to a close, boats were no longer in use in the Thule region, and whaling was eventually discontinued, only to be re-introduced. In 1818, the explorer John Ross was accompanied by a fleet of whalers across the Melville Bay and eagerly noted the abundance of whales the northern end of the bay (Ross 1819); the arrival of Ross, professional whalers, and later expeditions were to change the Inughuit’s sense of themselves and their place within an expanding world of resources—soon including wood, iron, rifles, and so forth, and involving them in a monetary economy, in turn precipitating new hunting ventures. It has been suggested that bowhead whales were not hunted in Smith Sound after early 19th century (Stoker and Krupnik 1993, pp. 579–629), but industrial whaling continued in the Baffin Bay, where it peaked with catches of 1600 bowhead whales in 1820, after which it gradually declined until 1850, when it stopped. Still, explorers pushed northwards in Smith Sound, where they affected the socio-ecological system by bringing in new materials and new knowledge.

Along with the Late Dorset (c. 650 AD) and Early Thule Culture migrations (c. 1250 AD) from the Western to the Eastern Arctic knowledge about sources of copper, iron and soapstone spread, and the exchange and trade networks intensified (McGhee 1997). From the Greenlandic side of the North Water, meteoritic iron—found in the vicinity of Savissivik (see Fig. 1)—was exported to western Inuit, while copper made it the other way. The iron was hammered off the meteorites with hammer stones, and transported north- and westwards sometimes as far as 1000 km from the source (Appelt 2004).

The early Thule Inuit in Northwest-Greenland also had connections to the Norsemen, who settled in south Greenland in the early Middle Ages (c. 1000 AD, to disappear around 1500 AD) (Appelt 2004). The Inuit (of the Ruin Island phase) took advantage of Norse shipwrecks (or even raided Norse expeditions to the North Water), and between them, they probably traded walrus tusk and (Norse or telluric) iron (Sutherland 2000; Schledermann 2000). Even if it is difficult to know who actually travelled where, both parties have left their traces in each other’s settlements. This case from the Early Thule Culture underscores the fact that climate, landscape, animals, and people with differing technologies, modes of organisation, and hunting practices all contribute to the plastic architecture of the ecosystem, as perceived by humans in terms of a resource space.

## Ice and desolation: Politics of space

While traffic across Smith Sound was relatively frequent in the warm medieval period, it dwindled in the period from 17th to late 19th century—as did habitation in general during the cooling of the Little Ice Age as noted above. Norwegian Otto Sverdrup, on expedition in the High Arctic (1898–1902), found no living people in the Canadian High Arctic archipelago at all. He was deeply affected by the fact that he only found ruins, from the ‘time of the Eskimos’, suggesting that these people belonged to a distant past. The discovery underscored the unimaginable exposure to a hostile nature that would have been their plight. Sverdrup writes how they were “gripped by a strange feeling of abandonment and waste when seeing these ruins, telling us that even here humans have lived with their sorrows and pleasures like us” (Sverdrup 1903, II, pp. 275–276; our translation).

The sense of Ellesmere Island as inhospitable to humans seems also to have been part of local knowledge in Thule. Jean Malaurie, a French geographer residing in and exploring the region 1951–1952, relates how his wish to cross over Smith Sound was met with a lot of scepticism. At the defection of a young hunter an old man said:

We must have somebody young with a little blood in him. It’s not going to be easy if we’re going right to Canada. We shall certainly not have enough food. And I do want to come back. You know the story of the Americans? There with Grileysouak (Greely), they all died of hunger and devoured one another. And then if one has to pass the winter in the desert island of Ellesmere the cold is terrible. Ouloulik said that the petrol freezes there. If the sea-ice breaks between Canada and Greenland—and it will break perhaps—what will you do? We shall have to live by hunting. But you can’t carry a year of cartridges. So we shall have to use harpoons. (Malaurie 1956, p. 124)

The old man certainly had little faith in the expedition and resented being thrown back on old technologies, but eventually, it turned out to run smoothly; there were, indeed, plenty of seals on the other side. Yet for Greely, setting out towards the Polar Seas in 1881 and stranding on Pim Island (see Fig. 1), where eventually 19 of the 25 men died from starvation, there was no such luck on the coast (Dick 2001, p. 188ff).

It does take a fine-tuned attention to the High Arctic environment to survive; but even so, game is not a given. The meagre vegetal resources even in the most fertile valleys on which muskox (*Ovibus moschatus*) and caribou (*Rangifer tarandus*) would forage all too easily disappear, if not through grazing then under a deep cover of snow or through sudden freeze-up in the fall or an early thaw and freeze during winter/spring. Nevertheless, on Ellesmere Island there are also sheltered inland valleys with an unusual level of growth for this latitude (Dick 2001, p. 33), providing a habitat for the muskox, in some periods being a major attraction of Ellesmere Island for Inuit and Inughuit hunters in the Smith Sound region. Radiocarbon dating of muskox remains in Northwest Greenland has indicated that this species probably entered in the late Holocene c. 100–900 AD (Bennike and Andreasen 2005), perhaps sometime during the centuries when Greenland was uninhabited by humans (c. 100 BC–650 AD).

Politics also affects the making and, indeed, the unmaking of a resource landscape. Around 1900, Canada turned its attentions towards the Arctic Islands; Britain had transferred these islands to the dominion of Canada in 1880, but nothing much had happened. Yet, Robert Peary’s and his helpers’ repeated excursions from Greenland to Ellesmere Island during his long quest for reaching the North Pole (1891–1909), effectively reminded the Canadian authorities about their High Arctic islands. Coming from the Danish side, Peary inadvertently contributed to a potential Danish claim to the region, and the Canadians had to act, recalling how Sverdrup had once planted the Norwegian flag there (Dick 2001; Schledermann 2003). It was not enough to assert sovereignty; it also had to be demonstrated through occupation, and the Canadian authorities sat out to do just that. In hindsight, we understand that international exploration and science contributed immensely to the making of the Canadian Arctic (Levere 1993), first by navigating and mapping it, next by compelling the Canadians to invent ways of asserting their rights also to the islands along the coast of Smith Sound, affecting the resource-space of the inhabitants of Northwest Greenland.

Part of the concern in the early 20th century was the muskox that had been intensely exploited by the many recent expeditions. The emissary to Ellesmere Island for the Canadian government estimated that Peary and other American expeditions alone had taken 800 muskoxen over a generation; Sverdrup was presumed to have killed some 200 animals on Ellesmere Island during his sojourn there, while Cook had taken 100 during his polar expedition in 1908 (Dick 2001, pp. 268–269). This, it was feared, would have given the Greenland Inughuit a renewed taste for muskox, and the Canadian emissary advocated regulations “to prevent numbers of Eskimo natives of foreign countries exploiting Canadian territory, and destroying valuable hunting and fishing grounds” (Bernier 1910, cited in Dick 2001, p. 270).

Knud Rasmussen’s plans for the Fifth Thule Expedition apparently also alarmed the Canadians, hearing about them in 1920 and believing that he wanted to establish trade stations in Canada. He was told not to hunt muskoxen, to which he replied: “There is no question of our breaking Canadian Game Laws, because we are not coming into Canada but a part farther north. It is not under Canadian jurisdiction” (Rasmussen, cited in Dick 2001, p. 274). The latent conflict continued and in 1921 the Dominion Commissioner of Parks, J.B. Harkin, pressed on to make Canada curtail muskox hunting on Ellesmere Island by the Inughuit. He recommended

… that Canada should in the first place take a very strong stand in regard to its exclusive ownership of and authority over Ellesmere Land; that the Danish government should be advised that a continuance of the slaughter of musk ox in Ellesmere Land cannot be tolerated because it will mean the early extermination of the musk ox; that if Denmark will not immediately agree to entirely stop this slaughter Canada should establish a mounted police post in Ellesmere Land for the purpose of stopping the slaughter and asserting Canadian authority. (Harkin, cited in Dick 2001, pp. 274–275)

Such posts were eventually established in Craig Harbour (1922) and at Bache Peninsula (1926), ironically calling on the Inughuit from Northwest Greenland for help with transport and hunting—as there were no ‘Canadian’ Inuit in the region at all. This was to change during the Cold War, when Canada was requested to survey Ellesmere Island by the US, busy at establishing the Distant Early Warning (DEW) Line and wanting to extend it to High Arctic Canada (Doel et al. 2014). Consequently, and once again in the interest of protecting Canadian sovereignty, the Royal Canadian Mounted Police (RCMP) detachments on Ellesmere Island were to be re-established and ‘native’ settlements created—first at Craig Harbour along with the RCMP–post (1951) and later at Resolute and Grise Fjord (Dick 2001, p. 426ff).

In his comprehensive description of the Thule District from 1921, Knud Rasmussen does not include the western side of Smith Sound directly in his description, which is structured as a travel account from South to North along the coast from Cape Melville to Inglefield Land, which was after all what constituted the District. Yet in his description of daily life and seasonal hunting practices, he mentions the need for bearskin, not only for clothing but also for platform cover “now the reindeer are almost extinct”. He adds that not all are equally efficient bear-hunters, which is why people have begun to hunt muskox even on the far side of Ellesmere Island, to procure the hides (Rasmussen 1921, pp. 561–562). He adds:

As such a muskox hunt may extend over 6–8 weeks, especially if they go all the way to Heiberg’s Land, sometimes the wives are also taken along. The hunters then camp at the hunting grounds to dry the skins. Every year, one may count on some twenty hunters to engage in this hunt. Estimating that every year some 300 muskoxen are killed, it is to be sadly expected that it only is a question of time when this peculiar big game shall be expelled from or extinct in this region. (Rasmussen 1921, p. 562; our translation)

There is little doubt that the Inughuit at this time saw Ellesmere Island as part of their hunting grounds, offering particular resources where others had dried up. Apart from the muskox, they also had other inducements to crossing Smith Sound for hunting from time to time. The Danish biologist Christian Vibe noted how, in 1939–1941 when he stayed in the district, the Inughuit would make regular excursions across Smith Sound for polar bear, taking 10–20 or more on every trip (Vibe 1950, p. 95). He also observed how the ringed seals on the Greenlandic side were relatively small, while large seals were to be found on the coast of Ellesmere Island. “The old seals could keep open their breathing holes in the heavy ice more easily and stayed where they were, and the polar bear stayed with them” (Vibe 1967, p. 54). Clearly, it would have been attractive to go for the seals on Ellesmere Island, especially if it could also yield polar bear.

The history of habitation and exploitation of resources on Ellesmere Island shows how the ecological system, within which social life unfolds, is impacted by the emergence of new resources. In other words, and as suggested in the introduction, the ecosystem incorporates human practices and preferences, as well as political decisions and power struggles. These may operate at other time-scales than natural drivers, but their contribution should not be overlooked. Also, resources are not simply natural; they emerge as such within a social space that may expand or shrink in response to necessity and perceptions of resources, and of politics that restrict mobility. The value ascribed to particular resources is dependent on specific historical, economical, and social events. What is more, the ascription of value to particular species, such as the muskox, within a given *social* system potentially affects the entire ecosystem, of which both animal and human life is part.

Equally important to note are the different time-scales that adhere to the coastal regions around the Smith Sound, becoming part of its shifting contours. Biological, social and political forces conjoin and reshape the lived worlds in an uneven cadence. The composite agency of such forces makes the development of the ecosystem difficult to predict.

## New people and new materials: Eco-systemic effects

Human agency within the ecosystem and its interlocking temporalities is further illustrated by the arrival of two new groups of people in the Thule Region who had a major impact on the development of Inughuit life and outlook. They were European sailors and explorers, arriving from 1818 and onwards through the 19th century, among whom was John Ross, mentioned above, and a small group of Inuit Baffin-landers, arriving in the mid 1860s. Ross immediately began to speculate about the possibility of trade or an exchange of goods; for skins the newly discovered people could procure iron, wood, utensils, and later rifles and much more. (Ross 1819, pp. 119–120; Hastrup et al. 2018). The Baffin-landers were also to contribute vitally to the technologies of the inhabitants of Thule.

The Thule maritime hunting technology that had been introduced around 1250 AD changed remarkably during the beginning of the Little Ice Age. First, hunting of the bowhead whale ceased. The large whale harpoons and the *umiat* (sg. *umiak*) went out of use at least as whaling boats. Later, following a period of isolation and a diminishing of the Inughuit population, kayaks, and other technologies requiring wood, were not built anymore and thus the entire open water complex, including the throwing harpoons, had disintegrated well before the 19th century. Marine mammals were only hunted where they could be reached with thrusting harpoons from the ice and land. This was the material culture documented by the first 19th century explorers in Smith Sound (e.g. Ross 1819; Kane 1856; Hayes 1866). Hayes, in particular, notes the ingenuity of the walrus hunt, given the limited weapons and the absence of boats.

The immigration of a small group of people from Baffin Island to Smith Sound in 1864 changed the situation (Mary-Rousselière 1980). Many of them returned home after some years, but some stayed; five were still living among the Inughuit, while Peary was there (Peary 1898, I, p. 488). These newcomers had re-introduced the kayak and the open water harpoon hunting, greatly extending the reach of the hunter’s throw. A few of the remaining Islanders arriving in the 1860s were still alive, when Knud Rasmussen first came to Thule in 1903, and they could tell how they had taught people to use the (now) available wood for bows and arrows, fishing spears and not least kayaks (Rasmussen 1908, pp. 32–33; Hastrup et al. 2018). This brought back vital elements in their original technologies of movement and hunting.

With the later establishment of the Thule Station (1910), a new and steady supply of wood, rifles, and other materials, became available to the population, by then counting some 250 people (Gilberg 1976). A monetary economy was introduced and sledges and kayaks could be restored when needed, guns made the hunt easier, while metal-ware for pots and pans, cloth and sewing materials changed age-old routines of cooking and clothes-making. Hunger was generally curbed. Even epidemics began to abate, due both to growing resistance and to vaccination schemes, and on the whole, the Thule Station contributed vastly to a new sense of the community and the value of the hunt. However, the notion of value had shifted to a complex matter of money and new technological cravings. In addition to the steady supply of rifles, some people managed to get small motorboats, others had better houses—with walls covered in plywood, and in one case even sporting a wooden floor in the stone and turf house—much to the pride of the then young daughter (authors’ interviews in Qaanaaq 2010).

Clearly, migration and exchange of materials as well as new and incoming technologies and monetary value of hunting products affect the ways in which natural resources are perceived and exploited. So do the changing historical realities in (and beyond) the region. These may be commercial or political, and sometimes both, as happened with the establishment of the Thule Station in 1910, redefining the lived space and eventually the ecology of the place and its multi-species community.

## Changes in the ecosystem: Commercial drivers

The Thule Station was geographically situated in the Middle district, and it gradually evolved into a social and political centre in an otherwise decentralised society, also, as it happened, hosting several scientific enterprises over the years. As a trading post, the station facilitated an exchange of local goods, mainly fox fur and eider down, for foreign commodities, guns, clothes, coffee, etc. Fox skin trade had a major economic significance in the entire period of the Thule Station (1910–1953) and beyond; fox skins were traded until 1963.

The elderly inhabitants in the region still remember an unpleasant thing about fox hunting in the period, as it gave rise to a new kind of inequality among families; hunting fox for the skin and trading them for cash or commodities meant becoming involved in a new kind of economic order, differentiating between people and between places (authors’ interviews in Qaanaaq 2013). Some successful fox-hunters might suddenly become rich—and where sharing the hunt had been a tacit part of the social economy, sharing the money was not a given. The social consequences were not immaterial; in one case, it entailed such isolation of a very successful hunter that his surviving daughter (now well into her eighties) is still lamenting the unfairness of it. This has a parallel in the modern trade (including export) of *mattak* from narwhals (*Monodon monoceros*), where some may get rich from selling it, while others may only taste it at more or less communal events of celebration, when the lucky hunters—or those who had money to buy for before the *mattak* would leave the region—will take it out of the freezer and offer it up to the community, along with other delicacies.

Today there is no commercial fox hunting left. A small amount of cash is paid by the municipal office for the exchange of one fox-tail, as a measure taken to keep the population down, due to a general fear of rabies. The trade in fox skin was stopped by the Royal Greenland Trade Department due to a market collapse. The resettlement of the Thule population in 1953, moving the majority of the people northwards, much further from the fox-rich valleys around the dump at Thule Airbase, and on the coast between Thule and Cape York, where they could forage on little auks, also added a new spatial obstacle to hunting for people, who were not in possession of the necessary, strong motorboats. On the slope of Qaanaaq, chosen as the official replacement site of Thule in 1953, when people had to leave the Middle District due to the establishment of the American Airbase, foxes (*Vulpes lagopus*) are few and far between, because there are no seabird colonies close by on which the fox can prey (Mosbech et al. 2018).

A specific Thule-law was made in 1929, on the initiative of Knud Rasmussen, the owner of the station (see also Andersen et al. 2018). With this law, a hunters’ council was established with three representatives from among the hunters, from the North, the South, and the Middle district respectively, in addition to the residing doctor, the station manager, and the priest. One of the main objectives, apart from signalling ‘civilization’ by emulating international trends, was to regulate the hunt. It became increasingly clear that the process of centralization that had followed in the wake of the establishment of the Thule Station was beginning to exhaust the living resources in the Wolstenholme Fjord, notably walrus and eider duck, even if the semi-permanent population at Thule only counted some one hundred people (Holtved 1967). The walrus had suffered significantly from the introduction of firearms (Born 2005; Born et al. 2017). The hunters’ council urged able-bodied people to regularly disperse, as they had done before, and ruled that it was inadmissible to stay for more than 3 years in a row at Thule, unless people were sick or too old to move, in which case they would be taken care of by the Station. Spatial dispersal was one way of protecting the game, restrictions on the hunt was another. The preamble to this part of the Thule-law is significant:

Any free hunter may provide food and hides for himself and his family through hunting. But the game is no longer available in unlimited numbers. All over the world, independent people have therefore decided that the game animals must be protected at those times of the year, when they are breeding, because there shall otherwise be less and less game for every year. In our land, it is particularly important to protect eider ducks, foxes and walruses against extinction, and any free hunter should be pleased to go along with such protective measure, because these animals otherwise would be extinct, when those people, who are children now, become adult. (*Thule*-*law* 1929, p. 16)

The law further stresses that if the hunters do not comply with these measures of protection they will not only hurt the present generation but also the following generations. To further encourage people to disperse and thus to redistribute the hunt in the region, two auxiliary shops in the northern and the southern regions were established, in 1929 (Siorapaluk) and 1934 (Savissivik) respectively, creating new, if relatively minor centres in the region that again served as points of commodity exchange. This affected the spatial practices of hunting within the ecosystem—and hence its animal population.

Fishing has always been a potential resource in the area. In summer, when children are out of school, occupational and part time hunters bring along their families to favoured hunting sites throughout the area, where they hunt for narwhal, little auks, walrus, and fish for Arctic char (*Salvelinus alpinus*). The days, and sometimes weeks, in summer camps are treasured times where families spend time together and not only fill up their freezer but also gain strength for the cold and dark winter days ahead. Women in particular engage in the fishing of Arctic char by net from the beach; they make small camps and often see it as a kind of holiday from being a hunter’s wife (authors’ interviews).

Polar cod (*Boreagadus saida*) has always been fished by line from the ice, either by old men or women—who only need a small walking sled to cover the short distance to the crack in the spring-ice. Lately, the Greenland halibut (*Reinhardtius hippoglossoides*) has become of economic importance (Flora et al. 2018). We note how fishing—from being a task for women and old men (Arctic char, polar cod) is gradually becoming a modern industry (halibut) also attracting the men, fishing with long-lines from the sea-ice. Like the fox in earlier times, the halibut is becoming a significant commercial driver. This reflects the changing temperatures of the sea-water, attracting new species to the Smith Sound region.

While, generally, for people living at High Arctic latitudes (between 76°N and 79°N in the case of the Inughuit), hunting was always the essential survival strategy, it cannot be understood as a simple response to the presence of particular animals. Different resources have different values over time, depending on particular technologies and commercial interests as well as on availability of the animals and certain materials. Abundance of animals is again dependent on climatic fluctuations and on past histories of exploitation by humans in the Smith Sound region—or outside the region for migratory species. Hunting by foreigners of bowhead whales and walrus affected their availability to Inughuit: the development of garbage dumps at Thule Airbase increased the abundance of foxes; the re-introduction of reindeer and muskox (from South Greenland) has provided new resources; and the general warming of the Baffin Bay has likely increased the stock of halibut in Inglefield Bredning.

The main commercial drivers of hunting in Thule over the past 100 years were initially trade in fox skins, to be gradually replaced by trade in seal-skins until the seal-skin market collapsed in the 1980s, when it was replaced by trade in narwhal skin, which is still an important commercial driver. Generally, the living resources in Smith Sound have been ascribed changing commercial values at different points in time, in response to markets far beyond the region—be it of meteoric iron over half a millennium ago, or halibut fresh out of Arctic waters more recently. This means that the boundaries of the ecosystem have become ever more difficult to define, and that the human impact on the ecosystem has intensified—both locally and globally.

## The present ecosystem: Challenges and new possibilities in the thule region

Within Greenland, the community living by Smith Sound is one of the most persistent hunting communities. There are many hunters in Greenland, and quite a few towns and settlements where hunting is still essential for the economy, but in the farthest north and on the east coast, hunting permeates social life and its seasonality to the core. The hunt still embraces the biggest marine mammals like narwhals, polar bear and walrus, deeply affecting the self-perception of the people (Born et al. 2011, 2017). The hunting activities are not only a matter of light or darkness, or of the actual presence of the prey, but also a matter of ‘the right time’ for this or that hunt—under normal conditions. However, due to the general seasonal upheaval what used to be the ‘right time’ can no longer be relied upon, and in the field one senses the restlessness of people, who cannot access the polar bear (*Ursus maritimus*) or the walrus at a time when hunting is permitted, due to the changing ice-conditions. The hunt still dominates the perception of whom they, the Inughuit, are, and it is unsettling if the hunt fails, or if there is not enough polar bear furs to make trousers for both seasoned hunters and novices.

It is not only a concern for the (male) hunters. Women always played an important part in the processing of the catch, sharing out the meat, preparing the meals, processing the skins, and stocking up for the time to come. They also played a vital role in the making of clothes for the hunters (Holtved 1967). Although the identification with hunting is strong, things have also changed. Many women, especially those who are not married to hunters, have not learned these handicrafts. During the 1960s and 1970s, youngsters of both sexes were told that there was no future in the life as a hunter or hunter’s wife; formal education was a top priority in Greenlandic-Danish politics. Many girls were sent to Denmark for schooling and education, and did not learn the crucial handicrafts. One woman from Qaanaaq, now in her sixties, tells how she returned from schooling in other towns of Greenland and in Denmark, and expected that the peers who had stayed in Qaanaaq would all know the traditional handicrafts; to her surprise, only few had learnt them and valued other activities (authors’ interviews). Although hunting as a profession has regained value among the population in the Thule Region, many parents still emphasize that their children must get a formal education before choosing how to make a living.

The majority of young people leave Qaanaaq for formal education in Greenland or abroad at some point while becoming adults, and many never return. Further, the Greenlandic government is encouraging hunters to engage more in commercial fishing, materialised in significant subsidies on fishing equipment such as boats and outboard motors. While these subsidies become economic drivers for improving equipment and increasing fishing activity, the same equipment is used for hunting, and hence subsidies simultaneously become a driver for hunting of other game available. An interesting point to note is the relative stability of the number of registered occupational (full-time) hunters over the past decades, being some 80 persons. While hunters may occasionally re-define themselves as either full-time or part-time hunters (affecting their access to restricted game), and while the game may move and the hunt extend to halibut fishing, it seems that the hunting economy is sustainable at the level of some 80 full-time hunters, defined as such by their principal income, rather that by the species caught (and including fish).

**Fig. 2:**
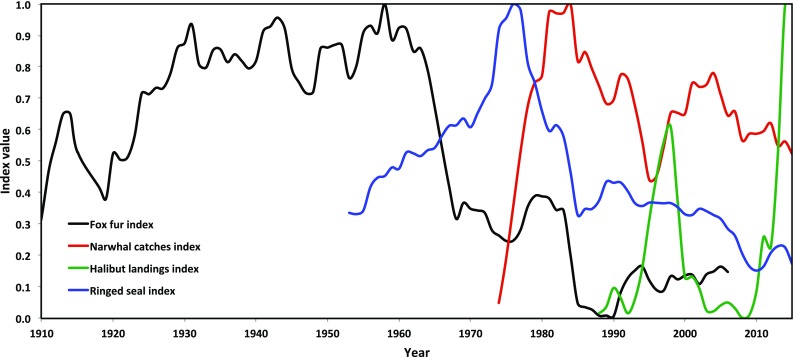
Development of catches of four cash generating game species utilised in the Thule Region from 1910 to 2015. Data for foxes and narwhals represent actual catches of individuals, whereas data on seals and halibut represent trade in seal skins and landings of halibuts, respectively. The data area scaled to proportions of the largest catch in the time series. The trends are shown as four-year gliding averages except for halibut where the few data only allowed for 2 year averages. Original data are from Statistics Greenland, Piniarneq and the Greenland Institute of Natural Resources

Two persistent modalities of the hunt, related to the seasonal contraction and break-up of the North Water ice edge give a first hint about the distinct technologies needed to access the game; first, the dog-sledge is essential to get to the ice edge, and to get to other places for other kinds of game during the season of fast ice. This season is shrinking, but the dog-sledge technology remains of paramount importance: it is the predominant technology of travelling. During the open-water season, the kayak and the motorboat replace the sledges. The kayak primarily plays a role in the hunt for narwhal, where use of motorboats is still prohibited, but also sometimes for open-water hunt of walrus. The price of narwhal-skin has increased precipitously since the late 1960s. With a present purchase price of up to 65 USD/kg and yield of on average 120 kg of skin per whale it is the most highly priced of all hunting products. As demonstrated above, when trade in fox skin ended, and when the international market for seal-skin had collapsed in the 1980s (Andersen et al. 2018), the narwhal became the game of highest commercial importance. Like the trade in fox fur did, the emergence of a market for narwhal *mattak* (skin and blubber) increased the monetary income among good hunters mostly on the Greenlandic market (see Fig. 2). As had happened with the fox-hunt, the commercialisation of *mattak* co-produced socio-economic differences within the Inughuit community.

Local practices support the sustained adherence to the kayak and the harpoon for the narwhal hunt. Hunters may set off in their motor-boat, but as soon as there are whales in the area they descend into their kayak and make the final, silent approach from the kayak. Harpooning directly from the ice-edge or, in rare cases from the beach, is also practised if the whale is close enough. Few whales are lost when using the harpooning-first-technique and kayaks are generally considered the best or only method for approaching the narwhals that are notoriously skittish and hard to get near in the Thule area. The rules for abandoning boat traffic in areas with narwhal hunting were originally introduced by the hunters’ council in the 1960s to protect the hunt, not the whales. The intention was to make sure that hunters without motorboats were not outcompeted and would still be able to catch narwhals without disturbance for hunters with boats. The rules are not formally included in the official rules for hunting narwhals in Greenland, but they are still widely practised in the Thule area.

The motorboat has become central for hunting in an increasing part of the year. Motorboats are less restricted by the ice floes than the fragile kayaks, so they fill in the gap between the overall use of the sledge during the period of fast ice, and the open water in the fjords when the kayaks are needed. Often, in the interim period, boats or kayaks are loaded onto sledges and brought out to the ice edge for hunting there. As some hunters, by virtue of subsidies from the Greenlandic government and income from the sale of *mattak*, steadily increase the horse-power of their outboard motors, the kilometres to far-away hunting grounds can be travelled faster than before. This extends the hunting area, while also complicating the relation between subsistence and commercial hunting by adding the layer of public subsidies. Along with increased hunting capacity, regulation of the hunt becomes more urgent.

In contrast to the highly specialised hunt for narwhals, the walrus-hunt is practised almost year-round, except for a few summer months, when the walrus prefers the cooler Canadian coasts (Heide-Jørgensen et al. 2017). In the spring, walruses are taken from motorboats that are ferried on dog sledges to the ice edge. Harpooning with floaters is practised from kayak when the animals are in the water, but walruses that are hauled-out on ice floes are often shot while asleep there. In the fall, long boat trips are made to meet the walruses that return from Canada to the northern part of the Smith Sound region near Etah, or to the area around the island of Kiatak on the outer edge of Inglefield Bredning.

With the introduction of a variety of new technologies, including motorboats, and new materials, including nylon cord or rope, plastic, and aluminium, the tools are gradually being refashioned, leaving out the skin of bearded seal, and walrus or narwhal tusk, in the hunting gear. Through the different hunting tools and items, and the way they integrate modern materials, the thrown-togetherness of the ecosystem, and the flexibility of society becomes tangible. Different materials carry their own economic and temporal dynamics in terms of acquiring them and making them ready for crafting a given tool. Generally, tools and technologies mediate the relation between hunters and their prey, infiltrating the ecosystem that humans inhabit and co-constitute, and within which they focus on particular species at certain times.

## Conclusion: Towards new futures

There is nothing inevitable about social practices, including hunting, around Smith Sound. Nor is the development of the natural ecosystem simply mechanical. Human actions are never just behavioural responses to a shifty environment; they are deliberate actions, foregrounding some practices at the expense of others, and favouring some kinds of game over others. People hunt on the basis of particular visions of space and social value, in addition to the obvious need for food, and for materials needed for hunting equipment. The ecological space, within which the Inuit have operated since their arrival in the region, is likewise a far from stable framework for orientation. It is affected by changes in temperature and ice-cover, by shifting animal abundance, and by the arrival of new people with unprecedented explorative or commercial interests, tying the apparently isolated region up with expansive global interests and conditions.

Hunting practices have changed in the wake of such new arrivals, bringing with them new technologies and materials. Traditionally, almost all equipment for hunting and movement were crafted with materials derived from game and local raw materials. In that sense, there was a circular connection between hunting activities and the equipment. This circular connection has changed, since more materials and technological devices (high-powered motor-boats, nylon string, plastic, canvas, iron, etc.) have entered the lives of the hunters. With these new materials and technologies, money is increasingly necessary and central for hunting to take place. In turn, this favours the hunting of game that may be sold for cash, such as narwhal and halibut. The new technologies are partly necessitated by the changing environment, forcing the hunters to go farter afield for game, and unable to do so by sledge—due to the shrinking sea-ice. There is thus a mutual precipitation of warming waters, re-fashioned hunting grounds, new technological needs, and an intensification of the monetary economy.

Above, we have wanted to convey a sense of the Smith Sound region as an ecosystem within which human activities make a difference—however small the population may seem in this vast landscape. The plasticity of the ecosystem and the drivers that change social and environmental relations within the ecosystem, weave together changing opportunities for human and animal life, changing interests and notions of value, again testifying to the remarkable viability of the social community in the Thule Region (Hastrup 2017). The temporalities of these changes may differ, but all of them affect the ecosystem.

Meanwhile, the larger upheavals in the region that are induced by changing temperatures and dwindling ice cover trigger more or less alarmist theories about the region as the “Last Ice Area”, leaving people in the region around Smith Sound with diminished hunting opportunities and possibly prey to extractive industry and tourism (WWF 2012). Truly, the ice cover is changing as is the sea beneath it, but if the past is something to go with, the people of the Smith Sound region is resilient and flexible (Hastrup 2009). Climate may change, but the region will continue to provide resources on which people may rely.
